# Implications of bacterial exposure to reactive oxygen and chlorine species

**DOI:** 10.1128/iai.00010-23

**Published:** 2026-06-10

**Authors:** Gracious Yoofi Donkor, Jan-Ulrik Dahl

**Affiliations:** 1 School of Biological Sciences, Illinois State University 6049, Normal, Illinois, USA; University of California at Santa Cruz, Santa Cruz, California, USA

**Keywords:** reactive oxygen species, oxidative stress, antibiotics, antibiotic resistance, bacterial stress defense, reactive chlorine species

## Abstract

To inhabit aerobic environments, microbes must be able to cope with the reactive byproducts of aerobic growth. These toxic compounds, also named reactive oxygen species (ROS), damage a variety of cellular macromolecules, and thus demand universal adaptive responses in microorganisms. Beyond ROS, microbes must also contend with exogenous oxidants such as reactive chlorine species (RCS) and reactive nitrogen species (RNS). In the context of antimicrobial activity, discussions on exogenous reactive species have typically focused on pathogen clearance during the oxidative burst of activated leukocytes. However, the discovery that oxidative stress may contribute to the activity of bactericidal antibiotics presents new possibilities for targeting the antioxidant defenses of pathogens as an antimicrobial strategy for potentiating current antibiotics. Considering the emergence of antibiotic resistance in many clinically relevant bacteria, and the concurrent decline in antibiotic discovery efforts, such alternative treatment options are increasingly needed. In this minireview, we discuss the well-established pathways of oxidative stress resulting from endogenous and exogenous sources of ROS and RCS. We also contrast the macromolecular targets of ROS and RCS, as well as specialized detoxification mechanisms evolved by bacteria to minimize damage caused by each species. The implications of ROS/RCS exposure are then discussed in two facets: their contributions to antibiotic resistance and involvement in the activity of bactericidal antibiotics. Finally, we highlight antimicrobial strategies whose mode of action is proposed to depend on or involve ROS formation.

## INTRODUCTION

Molecular oxygen (O_2_) is the preferred terminal electron acceptor of the electron transport chain due to its high redox potential and is therefore an essential driver of cellular metabolism in aerobically respiring microorganisms (1). However, O_2_ can react with reduced electron carriers in the electron transport chain to form the highly toxic radical superoxide (O_2_^−^) and the non-radical hydrogen peroxide (H_2_O_2_), collectively known as reactive oxygen species (ROS) (2). While hydroxyl radicals are also categorized as ROS, they are generally formed when ferrous iron (Fe^2+^) is oxidized by hydrogen peroxide (3). Although ROS formation and detoxification typically reach a steady-state equilibrium, excessive ROS production can elevate ROS levels beyond this equilibrium, overwhelming cellular defenses and causing significant macromolecular damage—a condition termed oxidative stress. In microbes, ROS exposure can arise from endogenous sources and a variety of environmental and exogenous factors. For instance, activated host immune cells, such as neutrophils, release high levels of ROS/RCS to control bacterial colonization (4–6).

Extended periods of oxidative stress are detrimental for most organisms as ROS/RCS can oxidize many cellular targets, including (i) nitrogenous bases and ribose in DNA (7), (ii) iron-sulfur clusters in iron-coordinating enzymes (8–10), and (iii) proteins, the major macromolecular constituents of cells. This causes diverse cellular insults, including protein carbonylation, which can result in protein unfolding and aggregation, as well as in mutagenesis and DNA double-strand breaks (11, 12). Consequently, phenotypic outcomes of cells that experience oxidative stress are often pleiotropic.

To mitigate the presence and consequences of oxidative stress, microbes have evolved sophisticated mechanisms to detect elevated levels of ROS/RCS, initiate their detoxification to restore redox homeostasis, and repair oxidative damage of cellular macromolecules (13–15). These bacterial response and defense strategies were found to be critical for the survival of bacteria in the phagosome and, therefore, may shape host-pathogen interactions (16–18). The presence of functional oxidative stress defense systems positively affects host colonization by pathogens (19–21), emphasizing their importance for pathogenesis.

Considering their potent antimicrobial effects, it is not surprising that ROS and RCS have successfully been used in antimicrobial therapy (22). Antimicrobials, which can disrupt cellular redox homeostasis, have emerged as potential alternative treatments in infectious disease control (23). Likewise, compounds purported to induce ROS have gained significant interest as potential avenues for antimicrobial treatment strategies (24–28). While these compounds will most likely be subject to the classical issues of resistance development that presently plague conventional antibiotics, there are also potential toxicity concerns due to the non-specificity of ROS in general. This review will briefly discuss the sources and cellular targets of ROS and RCS and review their individual detoxification systems in microorganisms. We then discuss the proposed contributions of oxidative stress to antibiotic lethality and arguments raised against this hypothesis. Finally, we present novel antimicrobial strategies centered on ROS induction as potential antimicrobial treatment options.

## SOURCES AND TARGETS OF ROS AND MICROBIAL STRATEGIES TO DEAL WITH ROS

### Endogenous sources of ROS

**Fig 1:**
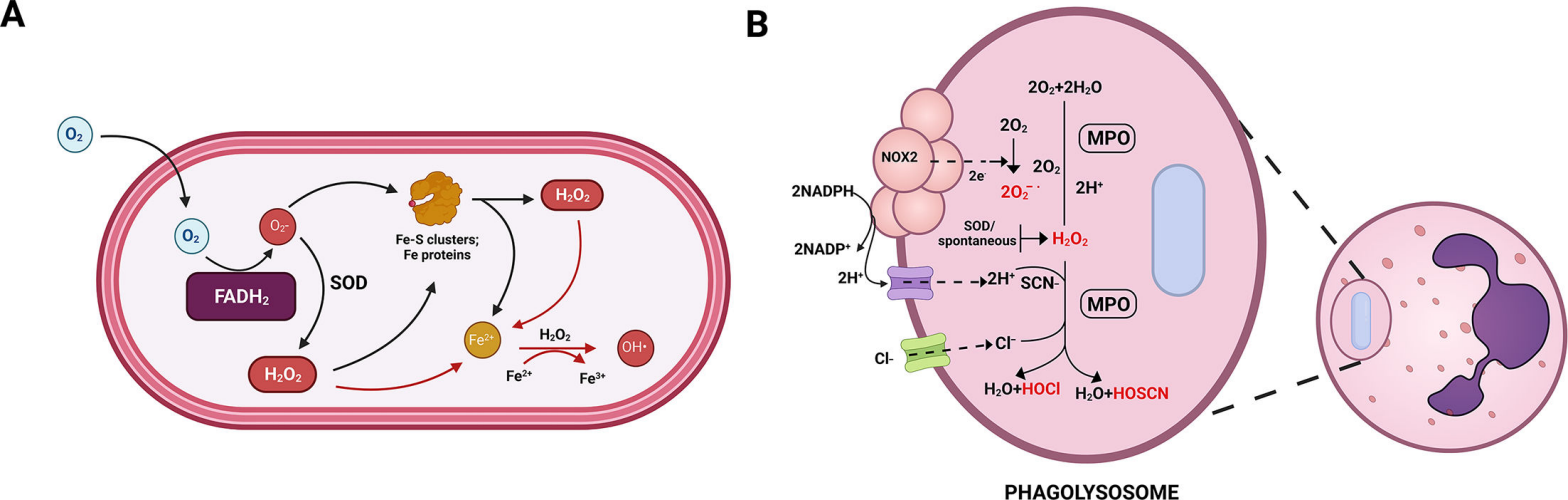
Overview of endogenous and exogenous ROS dynamics in bacteria. (**A**) Endogenous generation of ROS. The accidental electron transfer from reduced flavoenzymes to O_2_ results in the generation of superoxide (O_2_^−^), which can be converted into hydrogen peroxide (H_2_O_2_) by superoxide dismutases. O_2_^−^ and H_2_O_2_ can disrupt iron homeostasis by attacking surface-exposed iron centers of mononuclear iron enzymes and/or select Fe-S containing enzymes. The resulting increase in free iron results in hydroxyl radical (OH•) formation via Fenton reactions, leading to extensive macromolecular damage and ultimately cell death. (**B**) Exogenous ROS/RCS generation. Neutrophils kill invading bacteria in a process called oxidative burst, in which a cocktail of highly antimicrobial oxidants is produced in the phagolysosome. NADPH oxidase 2 (NOX2) located in the membrane of the phagolysosome catalyzes O_2_^−^ production through the reduction of O_2_. O_2_^−^ is enzymatically converted to H_2_O_2_, which serves as a substrate along with chloride and thiocyanate anions to produce the highly potent antimicrobial oxidants hypochlorous acid (HOCl) and hypothiocyanous acid (HOSCN), respectively. HOCl/HOSCN production is catalyzed by myeloperoxidase.

Due to its small size and non-polar nature, O_2_ can cross the cell membrane freely via passive diffusion (29). In the cytoplasm, O_2_ is consumed during oxidative phosphorylation to generate the free energy required to maintain a proton motive force (PMF) across the bacterial membrane. PMF is built up to drive ATP synthesis and facilitate other critical processes such as cellular transport and motility (30, 31). While respiratory enzymes such as flavoprotein dehydrogenases can transfer electrons to O_2_ during oxidative phosphorylation (2, 32), numerous other flavoenzymes present in abundance in the bacterial cytoplasm could contribute to the intracellular ROS pool. When O_2_ accidentally reacts with these reduced flavoproteins, the subsequent gain of a single electron generates O_2_^−^ and flavin semiquinone derivatives (Fig. 1A) (30, 33). The short-lived O_2_^−^ anions can be spontaneously or enzymatically dismutated into H_2_O_2_, a considerably more stable ROS (34, 35). The chain of ROS-generating reactions does not end here: O_2_^−^ and H_2_O_2_ can partake in additional ROS-generating redox reactions, for instance, by targeting surface-exposed redox-sensitive Fe-S clusters of dehydratase enzymes (36). Oxidation of these Fe-S clusters by either O_2_^−^ or H_2_O_2_ results in a less stable coordination of Fe(III) in the Fe-S cluster framework, leading to its disassembly, the release of Fe(III), which can readily be reduced to Fe(II) in the reducing environment of the cytoplasm (35, 36). O_2_^−^ and H_2_O_2_ can also oxidize the Fe(II) cofactors of mononuclear enzymes, compromising their enzymatic activity due to the dissociation of iron (37, 38). In subsequent reactions, Fe^2+^ can catalyze the conversion of H_2_O_2_ into highly reactive hydroxyl radicals (OH•), commonly referred to as the Fenton reaction (Fig. 1A) (36, 39).

### Exogenous sources of ROS

The generation of ROS is not limited to endogenous sources; microorganisms also frequently encounter ROS (and RCS, as discussed in the “Exogenous sources of RCS” section) from a variety of external sources. Intriguingly, the human host produces large amounts of these reactive species as a strategy to control a bacterial population. Dual oxidases (DUOX), a group of membrane-bound oxidases characterized by their transmembrane NADPH oxidase and extracellular peroxidase-like domains, transfer electrons from NADPH to molecular oxygen to produce H_2_O_2_ (40). These enzymes are expressed in several tissues and cell types, including mucosal barrier epithelia and uroepithelial cells of the bladder (41), where the generated ROS is required for controlling bacterial colonization of the epithelia (42, 43). Consistent with this, knockdown of the *duox* gene in the fly gut leads to a significantly increased rate of death due to a higher bacterial burden (44, 45). Colonization studies in DUOX knockout mice using *Helicobacter felis* showed an increased number of pathogens that colonize the intestinal epithelial cells (46). Moreover, innate immune cells, such as neutrophils, produce high levels of ROS to kill phagocytized microbes during oxidative burst (4, 6, 47). For instance, upon sensing foreign intruders, activated neutrophils undergo membrane remodeling to engulf the pathogens into internal vacuoles known as phagosomes (48), where activated NADPH oxidase located in the phagosomal membrane catalyzes the reduction of O_2_ into O_2_^−^, which dismutates spontaneously or is enzymatically converted into H_2_O_2_ (Fig. 1B). While H_2_O_2_ is generated in phagocytes in high amounts, kinetic studies indicate that in neutrophils, almost all H_2_O_2_ is sequestered and consumed by myeloperoxidase to produce hypohalous acids, which we will discuss further in the “Exogenous sources of RCS” section.

Plants, as well as many bacteria, release redox-active compounds, such as quinones and phenazines. These compounds can permeate target cells and undergo redox cycling, transferring electrons from redox enzymes to molecular oxygen (49, 50). A prime example is found in *Pseudomonas aeruginosa*, which produces phenazines to modulate its competitive interspecies interaction with *Staphylococcus aureus* (51). Furthermore, some lactic acid bacteria produce H_2_O_2_ as a byproduct of aerobic metabolism using flavoprotein oxidases such as pyruvate oxidase (52). Similarly, peroxide generation in lactic acid bacteria has been proposed to modulate interspecies competition (53).

ROS can also be found in diverse biotic/abiotic environments. In marine environments, dissolved organic matter can undergo photooxidation via solar radiation and enter an excited state, leading to O_2_^−^ production. O_2_^−^ is subsequently dismutated spontaneously into the more stable H_2_O_2_ (54, 55). Additionally, ROS are also generated at hydrothermal vents due to the abundance of H_2_S, dissolved O_2_, and trace metal catalysts, resulting in the formation of H_2_O_2_ and sulfide radicals (56). A similar reaction is proposed to occur at the oxic-anoxic interphase between the deeper lumen of the large intestine and the epithelial layer (57). Oxygen released into the subepithelial regions could react with hydrogen sulfide diffused from sulfate-reducing bacteria to produce H_2_O_2_ (57).

### Cellular targets of ROS

Exposure to ROS can induce covalent, irreversible amino acid side-chain modifications, resulting in the destabilization of protein structure and subsequent inactivation (58). Arginine, proline, and lysine side chains are particularly susceptible to irreversible carbonylation through metal-catalyzed oxidation, which is widely attributed to Fenton-type reactions in which enzyme-bound or protein-associated iron reacts with H_2_O_2_ to generate highly reactive OH• (30). Carbonyl derivatives of histidine and lysine can additionally be formed through secondary reactions with carbonyl groups present on oxidized proteins or other macromolecules. Due to their electron-rich sulfur groups, methionine and cysteine side chains remain the most vulnerable ROS targets (5).

ROS can also oxidize and damage nucleic acids. H_2_O_2_ and O_2_^−^ damage nucleic acids indirectly by damaging Fe-S clusters and increasing intracellular free iron levels, which can randomly associate with the DNA backbone (59). Subsequent reactions with local peroxide species can generate OH• radicals, which then oxidize nucleic acid ribose moieties (39). Due to their low reducing potential, guanine bases are preferentially oxidized, leading to the formation of the highly mutagenic 8-hydroxyguanine lesions (30), which can evade DNA proofreading due to their ability to pair with adenine.

While lipids are well-known targets of ROS in eukaryotes, it appears that lipid peroxidation in bacteria differs fundamentally due to the lack of polyunsaturated fatty acids (PUFAs), which are required to sustain the radical chain-propagation phase of lipid peroxidation (30, 60). A notable exception to this is seen in *B. burgdorferi*, where its ability to scavenge host-derived PUFAs into its membrane renders the pathogen vulnerable to classical, self-propagating lipid peroxidation (61). A similar observation has been made for *Vibrio* species, which accumulate PUFAs from the environment and integrate them into membrane phospholipids (62, 63). Nevertheless, studies have proposed that ROS and RCS can directly oxidize bacterial membrane lipids, leading to the formation of lipid hydroperoxides and secondary reactive aldehydes through non-propagative mechanisms (30, 60, 64, 65).

### Bacterial responses to and defenses against ROS

**Fig 2:**
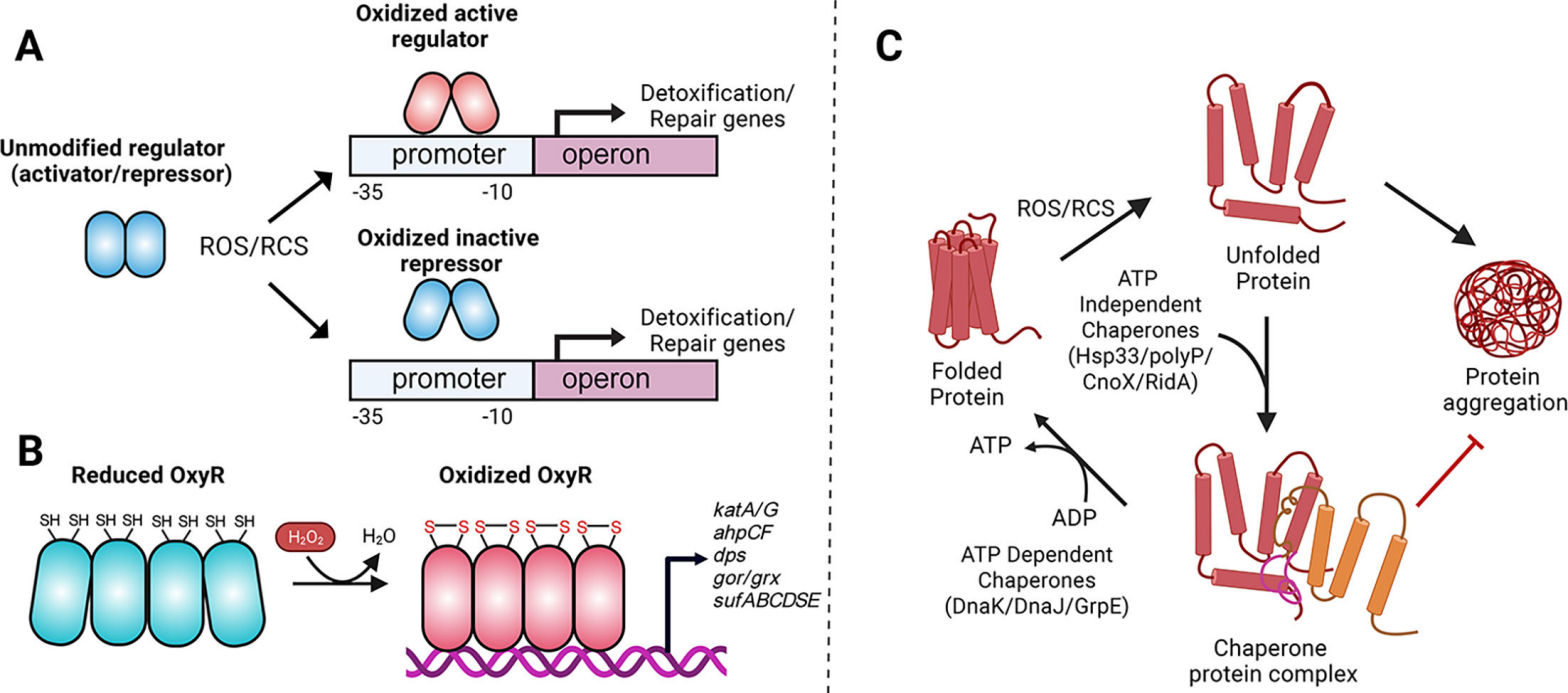
Bacterial mechanisms for oxidative stress defense. (**A**) Bacteria employ several redox-sensitive transcriptional regulators to regulate the expression of necessary detoxification genes in response to ROS/RCS stress. Redox-regulated transcriptional regulators are either activated or inactivated through reversible oxidation of cysteine and/or methionine residues, or Fe-S clusters, which alters their ability to bind to the target promoter and positively affects the expression of ROS/RCS detoxification genes. (**B**) OxyR represents a major transcription factor regulating defenses to ROS, respectively. Oxidation of thiol residues in the tetrameric OxyR protein causes conformational changes, which result in the upregulation of the OxyR regulon. (**C**) Following ROS/RCS-mediated damage of proteins, the most abundant cellular macromolecule, a repertoire of repair mechanisms is employed to reduce the risk of protein aggregation. Most prominently, molecular chaperones, such as Hsp33, RidA, CnoX, and Spy, become activated to stabilize unfolded proteins and prevent their aggregation. Following the removal of ROS/RCS, ATP-dependent chaperones initiate their refolding.

To deal with the consequences and eliminate ROS-mediated damage, microorganisms have evolved intricate systems to mitigate oxidative stress and repair macromolecule damage (recently reviewed in references 33, 66–70). One way for bacteria to respond to changes in their environment is by adjusting their gene expression, which occurs often because of the (in-)activation of oxidation-sensitive transcriptional regulators. Many of these redox-sensing transcription factors use conserved cysteine and/or methionine residues to modulate their activity and upregulate the transcription of their target genes, many of which have been shown to protect the organism from ROS and RCS (Fig. 2A) (71).

Bacterial responses to O_2_^−^ and H_2_O_2_ are mainly regulated by transcription factors such as SoxR, OxyR, PerR, and OhrR (30, 68, 72, 73). SoxR is a conserved MerR-family transcriptional regulator in *Enterobacteriaceae*, which coordinates the O_2_^−^ stress response (74, 75). Although the SoxRS regulon was originally believed to be induced by O_2_^−^, it is now established that SoxR can also be activated by redox cycling compounds and nitric oxide (75, 76). Upon oxidation of its [2Fe–2S] cluster, the SoxR homodimer undergoes conformational changes that allow SoxR to activate *soxS* transcription (77). SoxS, an AraC/XylS-type regulator, subsequently orchestrates a global response by inducing ~40 genes. These include *sodA* for superoxide dismutation, the *acrAB-tolC* efflux system for the removal of redox-active toxins, *zwf* to replenish NADPH pools, and *aroF* and *metE* for the biosynthesis of aromatic amino acids and methionine (78–80).

Responses to H_2_O_2_ are mediated by the LysR family-type transcriptional regulator OxyR. OxyR remains inactive at intracellular H_2_O_2_ concentrations below 50 nM (30). However, as H_2_O_2_ exceeds the threshold, OxyR’s conserved cysteine residues are reversibly oxidized to intramolecular disulfide bonds, which facilitate increased expression of the OxyR regulon (Fig. 2B). Members of the OxyR regulon include catalase, alkyl hydroperoxide reductases, and cytochrome c peroxidase (81), which collectively reduce H_2_O_2_ into water. To mitigate Fe-S cluster oxidation during H_2_O_2_ stress, the *suf* operon, another member of the OxyR regulon, is induced to compensate for H_2_O_2_-mediated inactivation of the housekeeping Isc system (82).

PerR and OhrR are conserved transcriptional repressors that contribute to bacterial responses to ROS/RCS stress but operate through distinct sensing mechanisms (71). PerR is inactivated by H₂O₂ via Fe²^+^-dependent histidine oxidation, which derepresses genes involved in peroxide defense and iron homeostasis. Although HOCl induces the PerR regulon, evidence suggests that this response is largely indirect, potentially mediated by HOCl-dependent inactivation of superoxide dismutase and elevated O_2_^−^ levels rather than direct RCS sensing (83, 84). On the other hand, OhrR is inactivated through cysteine oxidation, leading to derepression of the organic hydroperoxide reductase, OhrA (85, 86). While it remains unclear whether OhrR responds directly to RCS or to secondary organic hydroperoxides, loss of either *ohrR* or *ohrA* markedly increases sensitivity to both reactive species, demonstrating a direct protective role for the Ohr system (87).

Despite the significant toxicity of OH• radicals, no specific protein-based mechanism has been identified for OH• detoxification in microbes (13, 30). Rather, it has been proposed that OH• accumulation is prevented indirectly by limiting the availability of reactants required for OH• generation. For instance, the ferric uptake regulator (*Fur*), a global transcriptional regulator sensitive to intracellular iron concentrations, minimizes OH• formation by repressing iron acquisition systems. By maintaining a low labile iron pool, Fur prevents the sequestered iron from participating in the Fenton reaction (13, 88). Likewise, the iron storage protein Dps, a member of the OxyR regulon, has been shown to sequester free iron to limit OH• formation (89). Recently, YihE, a serine/threonine kinase, was found indirectly to limit the accumulation of OH• by inhibiting the MazEF toxin/antitoxin system, a mediator of programmed cell death in *E. coli* (90).

## SOURCES AND TARGETS OF RCS AND MICROBIAL STRATEGIES TO DEAL WITH RCS

### Exogenous sources of RCS

Oxidative burst in neutrophils does not end with the production of H_2_O_2_. Subsequently, the heme-containing enzyme myeloperoxidase converts H_2_O_2_ into hypohalous acids, including the highly potent antimicrobial oxidants hypochlorous acid (HOCl) and hypothiocyanous (HOSCN), respectively (Fig. 1B) (91). Recent work with genetically encoded redox probes showcased the severity of oxidative stress experienced by *E. coli* when trapped in the phagosome of neutrophils (92). The same study provided strong evidence that HOCl is the main component of the complex ROS and RCS cocktail produced in neutrophils. It has also been proposed that H_2_O_2_ and/or HOCl can react with Fe^2+^ to yield OH• in the phagosomal space (93, 94). However, this remains controversial given that lactoferrin, a component of the neutrophil secondary granules, would complex Fe^2+^ upon release into the phagosome and thus reduce its likelihood of reacting with H_2_O_2_ (38). More importantly, H_2_O_2_ would be rapidly consumed by myeloperoxidase to yield HOCl, being the more favored reaction (95). DUOX have been proposed to convert H_2_O_2_ into the potent antimicrobial oxidants HOCl as studies with HOCl-specific fluorescent probes revealed a dependence on HOCl production for DUOX-mediated bacterial killing (96). Finally, RCS can play an important role in defining symbiotic interactions, as seen between the Hawaiian bobtail squid and *Vibrio fischeri*. While the host’s generation of HOCl within the light organ renders the environment unfavorable to most microbes, *V. fischeri* has evolved specialized strategies utilizing a periplasmic catalase to actively suppress host RCS production. By inhibiting the respiratory burst and downregulating the squid halide peroxidase activity, *V. fischeri* effectively bypasses this oxidative challenge to establish an exclusive symbiotic niche, demonstrating the role of RCS in modulating host-microbe interactions (97).

### Cellular targets of RCS

Unlike H_2_O_2_, HOCl not only oxidizes proteins directly but causes significant protein unfolding and formation of toxic protein aggregates (58). RCS can inflict nucleic acid damage through a variety of direct and indirect mechanisms. HOCl can directly react with amino groups of nucleic acids to generate chloramines leading to DNA strand breaks (98). Additionally, HOCl can oxidize nucleic acid bases, for instance, oxidizing uracil groups to 5-chlorouracil (99). Due to its proteotoxic effects, HOCl also inhibits nucleic acid synthesis by disrupting protein transcription and translation factors (100).

### Bacterial responses to and defenses against RCS

It has become increasingly clear over the last years that bacterial defenses against RCS are not just extensions of canonical ROS stress responses, but instead involve dedicated sensing, regulatory, and repair pathways unique to RCS (33, 66, 67, 69, 101). While ROS responses often center on H_2_O_2_ detoxification and redox buffering, RCS elicit qualitatively different cellular damage, including rapid protein aggregation through thiol oxidation (102, 103), methionine sulfoxide formation (104), and protein chlorination (105–108), which require specialized adaptive strategies.

A defining feature of bacterial RCS defenses is the use of redox-regulated transcription factors that specifically sense RCS rather than ROS. In *Escherichia coli*, transcriptional regulators such as HypT (109, 110), the TetR-family transcriptional repressor NemR (111, 112), and the AraC-family transcriptional activator RclR (113, 114) respond preferentially to HOCl through either inactivation or activation, which occurs through oxidation of conserved methionines in HypT or cysteine residues in NemR and RclR, respectively. In either case, this leads to the expression of their target genes, which are largely distinct from classical OxyR- or SoxRS-dependent ROS regulons. In uropathogenic *E. coli* (UPEC), acquisition of the RcrARB system further illustrates pathotype-specific adaptation to neutrophilic stress, conferring specific resistance to RCS and neutrophil-mediated killing that is absent in intestinal strains (16). Importantly, this system does not provide broad oxidative stress protection but is highly selective for RCS. Growth comparisons of genome-sequenced clinical UPEC isolates at sublethal RCS stress revealed a strong correlation between increased RCS resistance and the presence of RcrB: HOCl resistance profiles of strains carrying the *rcrB* gene in their chromosome were all significantly more resistant than strains that lack this gene (115). The *rcrARB* gene cluster is regulated by RcrR, an RCS-specific transcriptional repressor under non-stress conditions (16). During RCS stress, however, as it occurs in the phagosome of neutrophils, RcrR is oxidized and forms oligomers through inter-subunit disulfide bond formation, leading to the inactivation of its repressor function and de-repression of the *rcrARB* operon. The resulting upregulation drives significant expression of RcrB, an inner membrane protein that has recently been shown to detoxify extracellular RCS through conserved redox-active residues, providing community-level protection (116). RCS-specific signaling is also evident in two-component systems, such as *E. coli* HprSR. Initially linked to H_2_O_2_, HprSR is now recognized to be more efficiently activated by RCS (117). Upon methionine oxidation of the HprS sensor kinase, the system induces the expression of *msrPQ*, a periplasmic methionine sulfoxide reductase system dedicated to repairing oxidatively damaged proteins within the cell envelope. Besides methionine sulfoxide reductases, dimethyl sulfoxide reductases have previously been identified as crucial protective players during HOCl stress (118). Collectively, these specialized sensing and repair pathways underscore the distinct mechanistic divergence between bacterial ROS and RCS stress responses.

Aside from time-consuming transcriptional adjustments discussed above, cells also have much faster-acting mechanisms in place to alleviate the deleterious effects of RCS, including the posttranslational activation of molecular chaperones. Numerous studies in different RCS-treated microorganisms revealed the strong upregulation of the heat shock regulon, indicating an accumulation of misfolded proteins during RCS exposure (Fig. 2C) and supporting the idea that proteins are major targets of RCS (102, 103). To mitigate the risk of protein aggregate formation, which is highly toxic to the cell, bacteria activate molecular chaperones, which also partially unfold during oxidative stress; however, in contrast to many other proteins, the loss in secondary structure leads to their activation (Fig. 2C). Following the removal of RCS, ATP-dependent chaperones, such as the DnaK/DnaJ/GrpE system, initiate client protein refolding. Several molecular chaperones have been identified that contribute to bacterial survival during RCS stress, including Hsp33 (119), CnoX (108), RidA (106), and, most recently, Spy (120). Hsp33 senses RCS or H_2_O_2_ in combination with elevated temperatures through four highly conserved cysteine residues in its C-terminal redox-sensing domain, which are rapidly oxidized during severe oxidative stress, resulting in Hsp33’s partial unfolding, activation, and binding to unfolded proteins to protect them from aggregation (121). In contrast, CnoX and RidA are activated through chlorination, while Spy activation relies on methionine oxidation (106, 108, 120). Aside from protein-based chaperones, Gram-negative bacteria convert intracellular ATP into polyphosphate, which contributes to protein homeostasis through its function as a molecular chaperone that efficiently prevents protein aggregation (122). Intriguingly, an FDA-approved drug was identified as a potent inhibitor of bacterial polyphosphate formation, sensitizing them toward RCS in various bacterial pathogens (20).

HOCl has also been shown to uniquely modulate protein activity. Proteins are the dominant cellular targets of HOCl, leading to rapid proteotoxic stress rather than the DNA damage typically emphasized in ROS biology. This distinction is exemplified by reversible HOCl-mediated inactivation of RecA (123) and activation of the diguanylate cyclase DgcZ (124, 125), linking inflammatory RCS exposure to DNA repair and biofilm formation. Detoxification strategies against the host-derived hypothiocyanous acid (HOSCN), which has distinctly different cellular targets compared to HOCl due to its thiol-specific mode of action (70, 126), rely on specialized NAD(P)H-dependent reductases such as RclA and Har (127–129). Intriguingly, the UPEC protein RcrB was identified as a novel RCS detoxification system that protects cells from RCS (116). RcrB acts as a membrane-associated, glutathione-dependent catalyst, intercepting RCS at the cell envelope and reducing oxidant influx. This reveals that effective RCS defense relies on localized, catalytic detoxification rather than bulk intracellular scavenging, which is a clever defense strategy considering the high reactivity and low specificity of RCS. In summary, several studies have established that bacterial survival in inflamed host environments depends on RCS-specific defense networks that are mechanistically and functionally distinct from canonical ROS responses. Bacteria have evolved coordinated and extensive defensive mechanisms that are highly attuned to sensing and mitigating ROS/RCS and/or the damage caused by these reactive species. Consequently, these defenses present an inherent challenge to ROS/RCS-dependent antimicrobials, as these protective networks must be overwhelmed or bypassed before a lethal threshold of oxidative damage can be achieved.

## CONNECTIONS BETWEEN OXIDATIVE STRESS AND ANTIBIOTICS

### The impact of oxidative stress on antibiotic resistance or tolerance

While it would be reasonable to expect at least an additive inhibitory effect when oxidative and antimicrobial stress are co-imposed, a few studies have reported the opposite. Increasing intracellular superoxide either through SOD-deficient mutant strains or via cotreatment with plumbagin, a redox-cycling naphthoquinone, antagonized killing by bleomycin (a metallo-glycopeptide) and reduced the inhibitory effects of different antibiotic classes (130, 131). Interestingly, antibiotic tolerance induced by oxidative stress was decreased in strains deficient in the transcriptional activators *soxS* and *marA*, as well as in strains lacking the efflux pump–encoding gene, *acrB* (131). Since the SoxRS regulon includes multidrug efflux systems, the O_2_^−^-induced tolerance was likely driven by efflux-mediated antimicrobial expulsion, induced by co-treatment with paraquat and plumbagin (131). The protective effect of O_2_^−^ exposure against antimicrobials has also been demonstrated under *in vivo* and *in vitro* conditions. For example, *Staphylococcus aureus* cells exposed to oxidative stress from activated neutrophils or the O_2_^−^-generating compound menadione showed up to 100-fold higher survival when subsequently treated with rifampicin (132). Furthermore, Gerstel et al. also uncovered that the redox-cycling compound phenazine-methosulfate increased fluoroquinolone resistance in *E. coli* by altering Fe-S cluster synthesis and activating the AcrAB efflux pump (133). These findings underscore a relationship between oxidative stress and the upregulation of efflux systems, as several SoxRS*-*regulated genes act in concert to reduce intracellular levels of redox-cycling compounds (134–137).

Similar dynamics have also been reported in response to other reactive species; in *Pseudomonas aeruginosa*, exposure to sublethal peroxide induced the expression of the *mexXY* multidrug efflux system via the oxidative stress-inducible *PA5471* (*armZ*) gene (138). Chronic peroxide exposure *in vitro* also selected for *mexXY-*dependent aminoglycoside-resistant mutants (138). Given that multidrug-resistant clinical isolates have been identified with constitutive *soxS* expression, antibiotic resistance could emerge as a consequence of chronic ROS exposure *in vivo* (139). The cystic fibrosis lung environment is known to be replete with oxidants due to chronic inflammation and imbalances in oxidant/antioxidant levels (140, 141). Indeed, hypermutable *Pseudomonas* strains harboring oxidative DNA damage have been isolated from the sputum of cystic fibrosis patients (142). These hypermutable strains had lower susceptibility to several antibiotics, including the membrane disruptor colistin and peptidoglycan synthesis inhibitor aztreonam (142).

Intriguingly, increased efflux pump activity supports the emergence of drug-resistant mutator phenotypes that show increased endogenous ROS levels (143). This phenomenon was observed in *E. coli* cells at the swarming edge, which exhibit high metabolic activity and increased antibiotic tolerance relative to their planktonic counterparts (143–145). In a previous study, a transcriptomic comparison of *E. coli* swarms and their planktonic counterparts showed that even in the absence of antimicrobial stress, swarming cells surprisingly upregulated genes encoding efflux pumps (i.e., *acrA*, *acrB*) and ROS detoxification genes (i.e., *katE*, *katG*, *sodB*, and *ahpC*) (146). Building on these observations, the authors showed that the drivers of this unique pattern of gene expression in swarming cells were linked to siderophore export, which is proposed to compensate for the low iron bioavailability encountered by advancing swarms on the agar surface (143, 147). The tradeoff for increased efflux was an increase in intracellular levels of ROS, detected via a redox-sensitive biosensor and ROS-sensitive fluorescent probes (143). *E. coli* swarm cells were also associated with decreased expression of *mutS*, *dinB,* and several other DNA repair genes (143). Not surprisingly, kanamycin-resistant isolates harboring defects in nucleotide excision and mismatch repair pathways were recovered from swarm cells. The emergence of these DNA repair defects appears linked to oxidative stress, as overexpressing *katE*, *sodB*, and *ahpC* decreased mutation frequencies in swarm cells (143).

### The contribution of oxidative stress to antibiotic lethality

Antibiotics have long been known to disrupt/inhibit bacteria-specific targets as the basis for their toxicity, including the disruption of DNA replication, transcription, ribosomal function, and cell wall biosynthesis. Aminoglycosides, for example, are known to exert bactericidal effects by targeting bacterial ribosomal subunits and promoting protein mistranslation (148). However, several studies, including a pivotal publication by Kohanski et al., reported that bactericidal antibiotics shared a common ROS-mediated killing mechanism in addition to their target-specific effects (149–153). This mechanism was proposed to involve ROS accumulation due to a hyperactivation of tricarboxylic acid cycle metabolic activity, Fe-S cluster disruption, and a breakdown in iron homeostasis (149). Utilizing hydroxyphenyl fluorescein, a fluorescent dye oxidized specifically by OH•, the authors discovered that only bactericidal antibiotics, such as aminoglycosides, beta-lactams, and fluoroquinolones, increased OH• detection, which was in stark contrast to bacteriostatic antibiotics (154). Interestingly, co-treatment of bactericidal antibiotics with iron chelators or the ROS-quenching agent thiourea substantially reduced killing and OH• levels (149). A follow-up gene expression study revealed an elevated expression of NADH-coupled electron transport genes (i.e., *nuoE*, *nuoC*, *nuoF*) under bactericidal antibiotic exposure relative to bacteriostatic antibiotic treatment (155). The authors also reported that bactericidal antibiotics inflicted DNA damage, as evidenced by the induction of the SOS response and the increased susceptibility of *recA*-deficient mutants to bactericidal antibiotic treatment (149, 155). The metabolic perturbations underlying the ROS-generating effects of bactericidal antibiotics were elucidated using systematic analyses of gene expression and phenotypic assays. Under aerobic conditions, aminoglycosides caused significant mistranslation and misfolding of membrane proteins, activating the CpxA two-component envelope stress sensor. CpxA activation was proposed to increase the activity of the TCA cycle through the activation of AcrA (155, 156). The proposed model suggested that these metabolic changes ultimately result in a hyperactive electron transport chain, increase endogenous O_2_^−^ and H_2_O_2_ levels, disrupt iron homeostasis, and thus accelerate cell death through Fenton chemistry (155, 157). The involvement of the TCA cycle in the ROS-generating qualities of antibiotics was also supported by other studies, including a study in *Staphylococcus epidermidis* where TCA-dependent ROS formation enhanced fluoroquinolone sensitivity. It was further reported that clinical isolates accumulate TCA cycle dysfunctions as a strategy to increase fluoroquinolone resistance (158). Treatment with beta-lactam antibiotics was also reported to induce ROS formation in *Enterococcus faecalis* (150). Although *E. faecalis* does not undergo TCA cycle metabolism, O_2_^−^ and H_2_O_2_ generation was found to occur via the autoxidation of the membrane-associated isoprenoid quinone demethylmenaquinone (150).

Despite the extensive studies proposing a role for ROS formation in antibiotic toxicity, this hypothesis remains highly controversial. A major point of contention is that anaerobically growing bacteria, which cannot form endogenous ROS, are still highly sensitive to bactericidal antibiotics (159, 160), further reinforcing the concerns about the general relevance of oxidative stress for antibiotic toxicity (161–164). Conflicting results have also been reported on the expression of ROS response regulons following exposure to bactericidal antibiotics (149, 165). The OxyR regulon was not significantly induced in independent transcriptomic studies of bacteria exposed to bactericidal antibiotics (149, 162, 165). Furthermore, the suitability of redox-sensitive probes often used to detect ROS in a variety of conditions, including antibiotic killing, has been questioned both with regard to their specificity and their sensitivity. A notable concern is that these probes can be oxidized non-specifically by factors such as heme enzymes and redox-active metals (166, 167). These concerns have recently been further supported by additional evidence from the Imlay laboratory (168), who also reported that the antioxidant thiourea may not be suitable for ROS quenching, limiting their use for detection of oxidative stress (169). Additionally, bacterial cells treated with bactericidal antibiotics exhibited higher autofluorescence, which could interfere with the redox probe measurements (163). The protective and OH• quenching effects of antioxidants identified against bactericidal antibiotics were also only relevant at low antibiotic concentrations (161). Additionally, the extent of antibiotic killing did not correlate with hydroxyphenyl fluorescein fluorescence (i.e., OH• detecting fluorescent probe), while bactericidal effects during anaerobic growth were only attenuated under aminoglycoside treatment and not in the presence of fluoroquinolones or beta-lactams (162). A similar study by Liu et al. also reported that three classes of bactericidal antibiotics showed significant killing in *E. coli* under anaerobic growth conditions. While anaerobic growth of *E. coli* decreased the lethality of the aminoglycoside kanamycin relative to treatment under aerobic conditions, the bactericidal effects of the fluoroquinolone norfloxacin or beta-lactam ampicillin remained largely unaffected (162). The decreased aminoglycoside lethality under anaerobic conditions was linked to a reduced PMF, which has been reported to be essential for aminoglycoside uptake (170). However, aminoglycoside uptake and subsequent membrane permeabilization have been demonstrated to occur even under diminished PMF conditions (171). The authors also reported that catalase- and peroxidase-deficient strains only showed increased sensitivity to norfloxacin but not to kanamycin or ampicillin. Additionally, perturbations in intracellular iron levels previously reported under treatment with bactericidal antibiotics were not corroborated, nor was a defect in DNA repair pathways detrimental to survival under antibiotic treatment (162). The protective effects of iron chelators and thiourea on antibiotic lethality were conferred under both aerobic and anaerobic conditions, potentially implying an ROS-independent mechanism (162).

Although the extent to which ROS directly contribute to antibiotic lethality is still disputed, Hong et al. have proposed a novel framework linking antibiotic killing mechanisms to oxidative stress (172). According to their model, antibiotic-induced ROS accumulation acts as a secondary amplifier of killing, particularly during the post-antibiotic recovery phase. Supporting this hypothesis, the authors demonstrated that bacterial survival increased ~30-fold when cells were washed to remove nalidixic acid (reducing levels to 0.002× the MIC) and then plated on LB medium containing the iron chelator dipyridyl compared to LB alone. While aminoglycoside tolerance due to dipyridyl has been linked to changes in PMF (164), this mechanism does not explain its protective effect against fluoroquinolones. Instead, the authors implicate decreased OH• formation due to reduced iron availability for Fenton chemistry as the primary mechanism underlying dipyridyl’s protective effect. Although these findings do not entirely prove a universal role for ROS in antibiotic killing, other studies found that inducing oxidative damage repair systems increases antibiotic tolerance, providing further credibility for a connection between antibiotic killing and oxidative damage (173). For example, Foti et al. observed that overexpression of MutT (which prevents incorporation of oxidized guanine nucleotides) and RibA (which enhances flavin-mediated antioxidant capacity) reduced bacterial sensitivity to multiple classes of bactericidal antibiotics (173). Although the relative contribution of these repair pathways compared to primary target inhibition by antibiotics remains unclear, their impact on survival indicates that oxidative damage may contribute to antibiotic-mediated cell death.

## EXPLOITING OXIDATIVE STRESS FOR ANTIMICROBIAL THERAPEUTICS

Antibiotic resistance has become a significant global health challenge due to the increasing emergence of multidrug-resistant isolates and a declining antibiotic development pipeline (174, 175). Antibiotic-resistant infections have emerged to all antibiotic classes and are projected to increase worldwide to 10 million deaths annually by 2050 (176). As the efficacies of current antibiotic regimens become increasingly limited by the spread of resistance mechanisms and the decline in novel antibiotics, ROS and RCS have gained interest as potential avenues in antimicrobial development (177, 178). While there are several antimicrobial strategies that have been proposed to either directly or indirectly center on ROS generation, a limited number of these are discussed in this review.

### ROS generating nanomaterials

**Fig 3:**
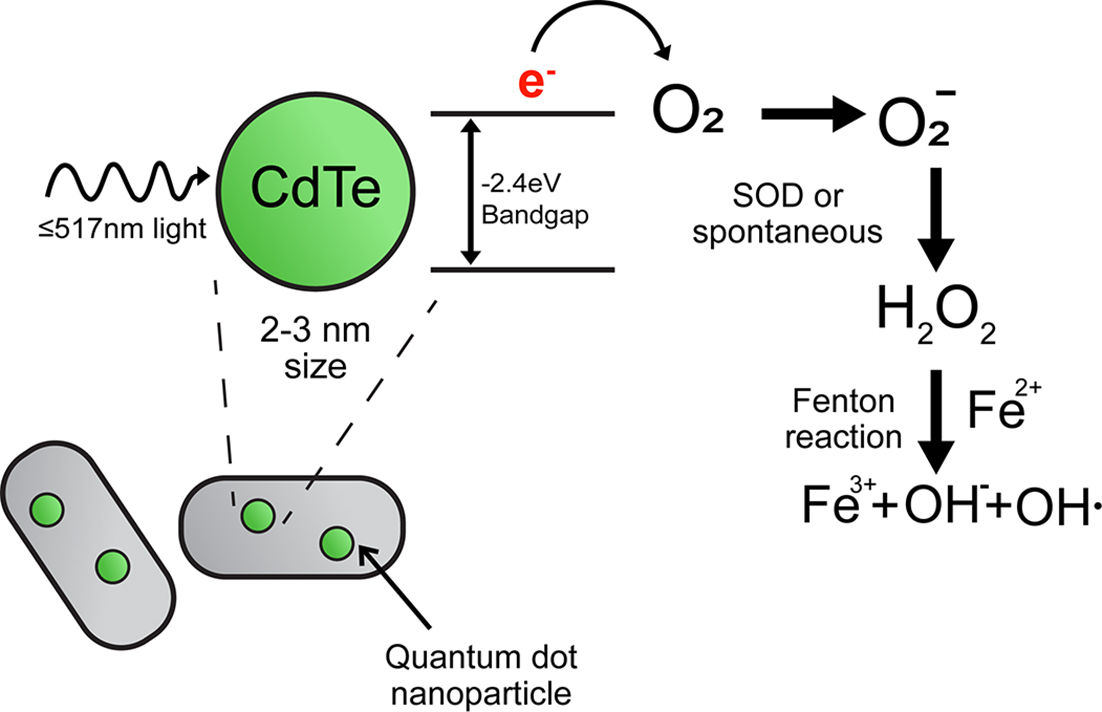
Proposed mechanism of action for superoxide-generating CdTe-2.4 nanoparticles. Exposure to visible light (< 517 nm) is proposed to excite electrons in the valence band into the conduction bands of internalized CdTe-2.4 particles, triggering the reduction of O_2_ to O_2_^−^, which can be enzymatically or spontaneously converted to H_2_O_2_. O_2_^−^ and H_2_O_2_ are proposed to attack vulnerable Fe-S cluster-containing proteins in bacteria, leading to an increase in intracellular iron. Increased ferrous iron is then proposed to react with H_2_O_2_ to generate the highly toxic OH•. In the absence of visible light, these reactions failed to occur.

Therapeutic applications for nanoparticles have been extensively investigated for applications in drug delivery and, more recently, for anticancer and antimicrobial therapeutics (179–181). Quantum dots (QDs) are nanoparticles synthesized from semi-conducting materials whose optical and electronic properties can be modulated for specific applications (181, 182). Several studies investigating QD antimicrobial properties report significant activity against laboratory strains and multidrug-resistant bacterial isolates (25, 182–184). QDs can be engineered by size and material to generate excited electrons at specific energy levels upon exposure to light. Excited QDs are then proposed to engage in redox reactions which can trigger O_2_^−^ production (Fig. 3). For instance, relative to treatment in the dark, cadmium telluride QDs activated with 2–4 nm light (referred to as CdTe-2.4 due to a 2.4 eV bandgap requirement for QD excitation) were demonstrated to show significant growth inhibitory and bactericidal activity against *E. coli* laboratory strains (25) (Fig. 3). CdTe-2.4 showed comparable antimicrobial effects in multidrug-resistant Gram-negative clinical isolates under similar conditions (25). To elaborate on their proposed redox-based mechanism, endogenous ROS formation was detected using the ROS fluorescent probe, 2,7-dichlorofluorescin diacetate (185), which was only detected upon activation with light (25). While the photo effect of CdTe-2.4 was attenuated under anaerobic conditions, the quantum dots retained substantial antimicrobial activity. This suggests that their efficacy is not solely dependent on ROS generation and points toward the existence of alternative, oxygen-independent killing mechanisms (25). CdTe-2.4 was also found to specifically generate O_2_^−^ and OH• radicals, as determined using spin-trapping-based electron paramagnetic resonance spectroscopy (26). Interestingly, supplementation with exogenous SOD quenched the O_2_^−^ and OH• signal generated by light-activated CdTe-2.4, while SOD-deficient and SOD-overexpressing *E. coli* strains were characterized by significantly increased and decreased growth inhibition upon CdTe-2.4 treatment, respectively (26). Intriguingly, increasing intracellular CdTe-2.4-induced O_2_^−^ rendered multidrug-resistant *E. coli*, *Salmonella enterica*, and *Klebsiella pneumoniae* more sensitive to certain antibiotic classes (26). Combination of CdTe-2.4 with both bacteriostatic and bactericidal antibiotics led to a synergistic increase in their antimicrobial activity against multidrug-resistant isolates in two-thirds of drug-CdTe-2.4 combinations tested. More importantly, a combination of the O_2_⁻-inducing nanoparticle with ciprofloxacin in a *C. elegans* infection model increased host survival relative to ciprofloxacin alone (26).

Although the antimicrobial activity of photo-activated QDs has been convincingly demonstrated, the question remained as to whether the nanoparticles could be specifically targeted against bacteria or implemented therapeutically with minimal collateral damage. To answer these questions, CdTe-2.4 activity was evaluated against *E. coli* in co-culture with HEK293T cells. While CdTe-2.4 completely inhibited *E. coli* growth, the viability and morphology of the HEK293T remained unaffected (25). The partial toxicity of CdTe-2.4 against bacterial cells was likely not due to specific/selective uptake into bacterial cells since QD nanoparticles can be internalized by human cell lines (186). It is more plausible that the low CdTe-2.4 quantity applied (35 nM showed significant activity against *E. coli*) was sufficient enough to inhibit bacterial cells but below the threshold required for HEK 293T cytotoxicity (25). However, uptake of QD nanoparticles into human cells could potentially become deleterious if they somehow accumulate within cells following repeated exposure.

### ROS induction as an antibiotic potentiating strategy

The discoveries concerning the synergistic effects of ROS generation on antibiotic activity provide some evidence that oxidative stress could potentially be employed to aggravate antibiotic lethality, at least against aerobically growing bacteria (187). Later studies have provided some confirmation on the possibility of increasing antibiotic potency by amplifying endogenous ROS stress. For instance, a recent study on antibiotic tolerance in *Mycobacterium* spp. provided evidence that a 20% drop in dissolved oxygen resulted in enhanced *Mycobacterium* persistence against the bactericidal antibiotics ciprofloxacin and isoniazid, respectively, while a commensurate increase in dissolved oxygen had the opposite effect (188). In a more expansive study, Brynildsen et al. utilized a flux-balance approach and generated an *in silico* metabolic network to predict genes whose deletion could increase endogenous ROS generation in *E. coli*. To confirm their *in silico* findings, the authors identified a number of genes whose deletion increased ROS levels *in vivo* and sensitivity to the H_2_O_2_-/ O_2_^−^-generating compound menadione (189). These genes mostly encode respiratory proteins, such as *cyoA* (i.e., subunit (II) of cytochrome b_0_ terminal oxidase), *nuoG* (i.e., NADH-quinone oxidoreductase subunit G), *sdhC* (i.e., succinate dehydrogenase cytochrome b556 subunit)*,* and *pta* (i.e., phosphate acetyltransferase) (190). More importantly, the authors reported that carboxin, an inhibitor of succinate dehydrogenase, increased susceptibility to both H_2_O_2_ and ampicillin, which may find its application as an adjuvant to potentiate antibiotic efficacy upon further development (190).

### Silver derivatives

Despite its long-standing history and high efficacy against bacteria, the antimicrobial mode of action of silver (Ag) is poorly understood. Pleiotropic effects have been described for Ag treatments and include changes in DNA condensation, membrane alteration, and protein damage (191). Ag interacts with cysteine thiols, destabilizes iron-sulfur clusters, and replaces metal-containing cofactors, which affects a wide range of proteins. More recently, Ag derivatives have received increased attention in medical applications, e.g., as antimicrobial surface-coatings on catheters that protect from biofilm-forming bacteria and reduce the risk of nosocomial infections (192). Moreover, Ag is used in topicals to prevent and/or treat infections in wounds (193). One such example is Ag-sulfadiazine, the gold standard for treating and preventing *P. aeruginosa* infections in burn wounds. However, Ag-sulfadiazine is associated with complications such as allergic reactions to the sulfadiazine moiety, emphasizing the need for novel treatment therapies (194).

**Fig 4:**
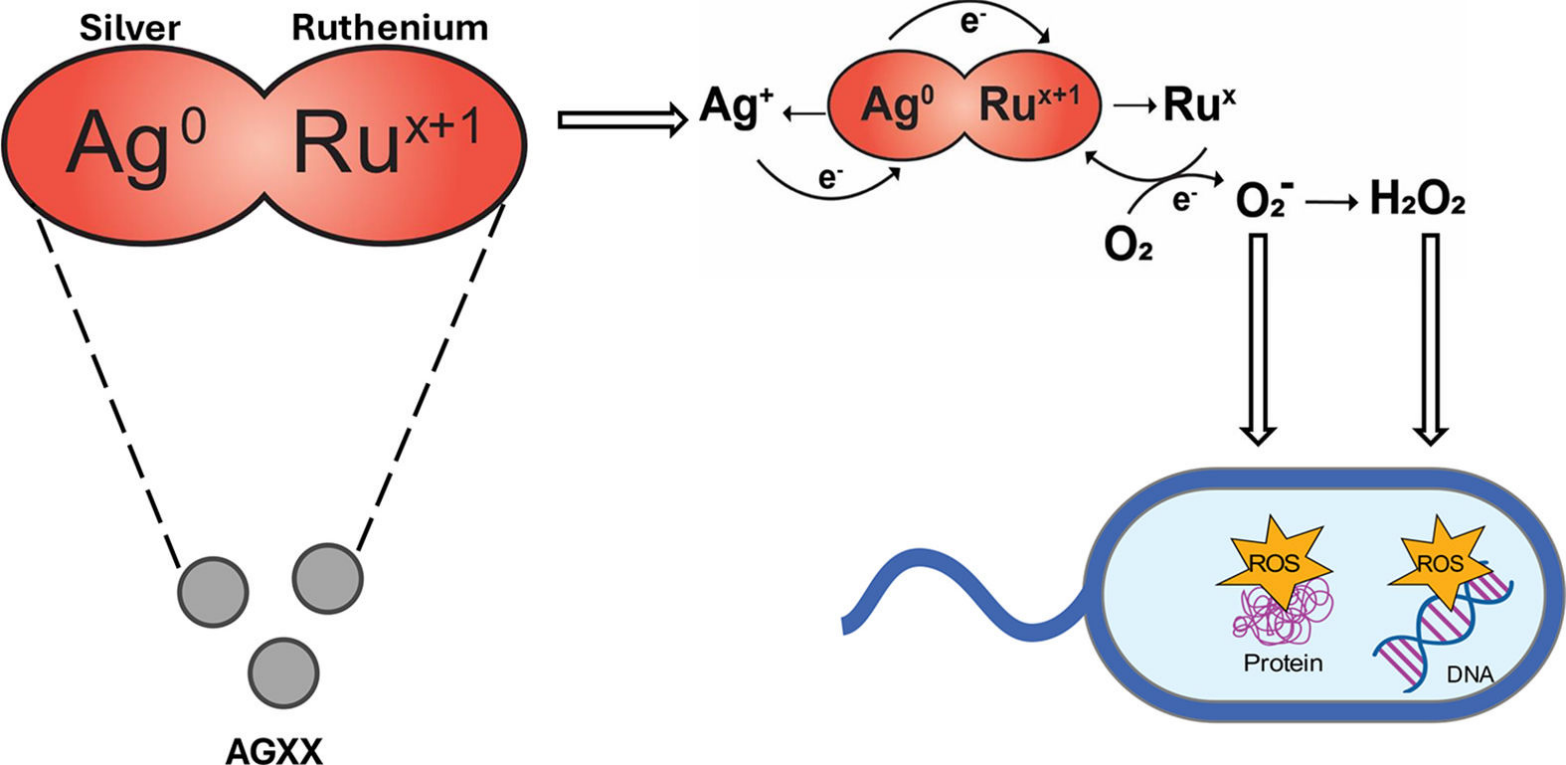
Proposed mechanism of action and downstream effects of AGXX. The novel silver-ruthenium complex AGXX, for which no resistance has been reported yet, generates ROS through the redox cycling between silver (Ag) and ruthenium (Ru) when in contact with organic matter, directly causing macromolecular damage that leads to bacterial cell death.

AGXX is a novel Ag-containing antimicrobial that was designed as a surface coating and is composed of micro galvanic elements of Ag and ruthenium (Ru) that are surface conditioned with ascorbic acid (Fig. 4) (195). AGXX inhibits the growth and biofilm formation of various antibiotic-resistant Gram-positive pathogens, such as *S. aureus* and *Enterococcus faecalis*, with significantly higher efficacy than classical silver (196). The antimicrobial spectrum of AGXX was extended to filamentous fungi and certain virusesand, most recently, also to Gram-negative bacteria (197–199). AGXX exerts its antimicrobial action through a catalytic redox reaction where Ag and Ru function as micro-galvanic elements to convert atmospheric oxygen into ROS, primarily H_2_O_2_ and O_2_^−^ (Fig. 4) (196). Ag and Ru serve as the anode and cathode, respectively, where Ag gains a more negative potential by reacting with chloride anions to AgCl, subsequently reducing via organic matter. This leads to the reduction of the higher valent Ru^x+1^ and the formation of H_2_O and H_2_O_2_. The authors further proposed that reduced Ru can be oxidized by O_2_ leading to an additional generation of O_2_^−^ radicals. The H_2_O_2_-generating property of AGXX was demonstrated using a photometric micro-detection method, where xylenol orange exposure to the Ru cathode of an AGXX galvanic cell detected H_2_O_2_ generation (196, 200). Through the release of Ag ions and subsequent mismetallation, AGXX potentially generates OH• in a Fenton-like reaction, further potentiating AGXX’s oxidant capability (98, 113). Ag and other soft metals have been documented to disrupt Fe-S clusters of dehydratase enzymes, increase intracellular iron pools, and facilitate the generation of OH• radicals via Fenton reactions (201). Transcriptomic studies of AGXX-stressed bacteria confirmed an oxidative stress-inducing mechanism of AGXX: AGXX treatment was marked by significant induction of oxidative and proteotoxic stress-response genes, including members of the heat-shock response and ROS-detoxifying antioxidant enzymes such as superoxide dismutase and catalase (196, 197). Likewise, AGXX treatment resulted in significant upregulation of H_2_O_2_ and, surprisingly, HOCl stress-response regulons in methicillin-resistant *S. aureus* (202). The de-repression of ferric uptake regulator (*Fur*) and zinc uptake regulator (*Zur*) regulons implied a disruption of metal ion homeostasis. A similar study in MRSA uncovered a significant downregulation of *agr* quorum sensing and biofilm regulating genes, indicating that AGXX could potentially attenuate virulence and biofilm formation in *S. aureus* (195). AGXX has recently been shown to cause substantial protein aggregation even when applied in sublethal concentrations, which likely contributes to its mode of killing (197, 203). Due to connections identified between ROS production and antibiotic lethality, a study by Donkor et al. hypothesized that AGXX could potentially increase the activity of conventional antibiotics. This and a follow-up study showed substantial synergistic effects between AGXX and aminoglycosides in various laboratory strains and clinical isolates and, in fact, restored the sensitivity of several multidrug-resistant bacteria (198, 199). Elevated ROS production was identified as an important contributor to the synergy, as the addition of ROS scavengers resulted in reduced endogenous ROS levels and increased bacterial survival. Likewise, strains deficient in ROS detoxifying/repair genes were more susceptible to AGXX/aminoglycoside co-treatment. This synergistic interaction was also accompanied by a substantial increase in outer and inner membrane permeability, which resulted in increased antibiotic influx and caused significant protein aggregation and DNA damage (198, 199). Since AGXX has no known resistance or toxicity to human cells (204, 205), it is an ideal antimicrobial candidate for surface coatings in medical applications. The need for improved surface coatings on medical devices to eliminate biofilm-forming pathogens via contact-killing is particularly pressing in light of the rise and spread of antimicrobial resistance (206, 207).

## CONCLUDING REMARKS

Investigating the dynamics of oxidative stress exposure in microbes is not only relevant for understanding the microbial adaptations evolved to ameliorate such stress but also for the effectiveness of antibiotic therapy. The dichotomous relationship identified between oxidative stress and antibiotic resistance seems to hinge on the spectrum of oxidant exposure severity, where low oxidant levels encourage resistant phenotypes, while more severe stress increases susceptibility to certain antibiotics. This presents a challenge to ROS-focused therapeutics due to the potential of adventitious development of drug tolerance or resistance following continued use of such antimicrobials. Longitudinal studies evaluating the impact of ROS-based antimicrobials on antibiotic resistance would perhaps be necessary to ensure this novel strategy does not compound the already pervasive challenge of antibiotic resistance. Additionally, extensive studies on their potential cytotoxicity are necessary since eukaryotic cells are susceptible to the deleterious effects of ROS. Nevertheless, the promising findings from studies discussed highlight several possibilities for disrupting redox balance in pathogens to eliminate them, sensitize them to otherwise obsolete antibiotics, or potentially sensitize them to immune clearance. Such applications would expand the repertoire of effective antibiotics against multidrug-resistant pathogens and potentially counter the emergence of antibiotic-resistant pathogens.
